# A drug target for erectile dysfunction to help improve fertility, sexual activity, and wellbeing: mendelian randomisation study

**DOI:** 10.1136/bmj-2023-076197

**Published:** 2023-12-12

**Authors:** Benjamin Woolf, Skanda Rajasundaram, Héléne T Cronjé, James Yarmolinsky, Stephen Burgess, Dipender Gill

**Affiliations:** 1 School of Psychological Science, University of Bristol, Bristol, BS8 1TU, UK; 2 Medical Research Council Integrative Epidemiology Unit, University of Bristol, Bristol, UK; 3 Medical Research Council Biostatistics Unit, University of Cambridge, Cambridge, UK; 4 Centre for Evidence-Based Medicine, University of Oxford, Oxford, UK; 5 Faculty of Medicine, Imperial College London, London, UK; 6 Department of Public Health, Section of Epidemiology, University of Copenhagen, Copenhagen, Denmark; 7 Population Health Sciences, University of Bristol, Bristol, UK; 8 Department of Epidemiology and Biostatistics, School of Public Health, Imperial College London, London, UK

## Abstract

**Objective:**

To investigate the association of genetically proxied (using a surrogate biomarker) inhibition of phosphodiesterase 5 (PDE5), an established drug target for erectile dysfunction, with fertility, sexual behaviour, and subjective wellbeing.

**Design:**

Two sample cis-mendelian randomisation study.

**Setting:**

Summary data on genetic associations obtained from the International Consortium for Blood Pressure and UK Biobank.

**Participants:**

Individuals of European ancestry from the International Consortium for Blood Pressure (n=757 601) for estimating PDE5 inhibition (using the surrogate biomarker of diastolic blood pressure reduction), and UK Biobank (n=211 840) for estimating the fertility, sexual behaviour, and subjective wellbeing outcomes in male participants.

**Intervention:**

Genetically proxied PDE5 inhibition.

**Main outcome measures:**

Number of children fathered, number of sexual partners, probability of never having had sexual intercourse, and subjective wellbeing.

**Results:**

Genetically proxied PDE5 inhibition was associated with male participants having 0.28 (95% confidence interval 0.16 to 0.39) more children (false discovery rate corrected P<0.001). This association was not identified in female participants. No evidence was found of an association between genetically proxied PDE5 inhibition and number of sexual partners, probability of never having had sexual intercourse, or self-reported wellbeing in male participants.

**Conclusions:**

The findings of this study provide genetic support for PDE5 inhibition potentially increasing the number of children fathered by male individuals. Absence of this association in female participants supports increased propensity for sustained and robust penile erections as a potential underlying mechanism. Further studies are required to confirm this, however, and these findings should not promote indiscriminate use of PDE5 inhibitors, which can also have harmful adverse effects.

## Introduction

Phosphodiesterase 5 (PDE5) inhibitors such as sildenafil, vardenafil, tadalafil, and avanafil are commonly used for the treatment of erectile dysfunction and pulmonary hypertension.^1^ PDE5 is an enzyme that promotes the breakdown of cyclic guanosine monophosphate in vascular smooth muscle cells. By inhibiting PDE5, increased cyclic guanosine monophosphate activity induces vascular smooth muscle relaxation and vasodilation. In the setting of erectile dysfunction, this increases blood flow to the penis to facilitate sustained and robust erections.^1^ In the setting of pulmonary hypertension, PDE5 inhibition induces dilation of the pulmonary vasculature and improves ventilation-perfusion matching.^2^

Although randomised clinical trials provide vital data on drug efficacy, safety, and adverse effects, the limited duration of use does not always permit investigation of longer term outcomes. For PDE5 inhibitors, longer term outcomes could include effects on fertility, sexual behaviour, and subjective wellbeing. As PDE5 inhibitors are available to buy over the counter in countries such as the UK, it is important to understand their potential application for improving fertility^3^ and wellbeing.^4^ It is feasible that facilitation of penile erections and resultant fulfilling sexual intercourse may simultaneously increase the probabilities of both conception and improved subjective wellbeing.

Investigating such effects using traditional observational studies is undermined by confounding from environmental factors and reverse causation. Mendelian randomisation is an alternative epidemiological approach for strengthening causal inference in observational study designs.^5^ ^6^ Mendel’s laws of inheritance state that genetic variants are inherited independently during meiosis and should therefore not systematically relate to environmental factors. In the mendelian randomisation paradigm, random allocation of genetic variants predicting a given phenotype at conception is analogous to random allocation to intervention on this phenotype in a randomised clinical trial.^7^ Furthermore, genetic variants are fixed at conception, which confers a greater robustness of mendelian randomisation studies to bias from reverse causation.

Given that most drug targets are proteins and that genes encode proteins, mendelian randomisation has been paradigmatically extended to study the effects of perturbing specific drug targets.^8^ In such drug-target mendelian randomisation studies, variants located at the gene encoding the protein drug target of interest, so-called cis variants, are used as instrumental variables for studying the effect of perturbing that drug target pharmacologically.^9^ Such cis-mendelian randomisation can provide quasi-randomised evidence for outcomes that might otherwise be impractical or unethical to investigate within a randomised clinical trial. For example, a recent cis-mendelian randomisation study investigated genetic evidence for the safety of two major antihypertensive drug classes in pregnancy.^10^

Given the known effects of PDE5 inhibitors on promoting sustained and robust penile erections, and the ability of this physiological state to facilitate fulfilling sexual intercourse, we hypothesised that PDE5 inhibition may have effects on male fertility, sexual behaviour, and subjective wellbeing. We therefore performed cis-mendelian randomisation to investigate associations of genetically proxied PDE5 inhibition with each of these three outcomes.

## Methods

### Study design

**Fig 1:**
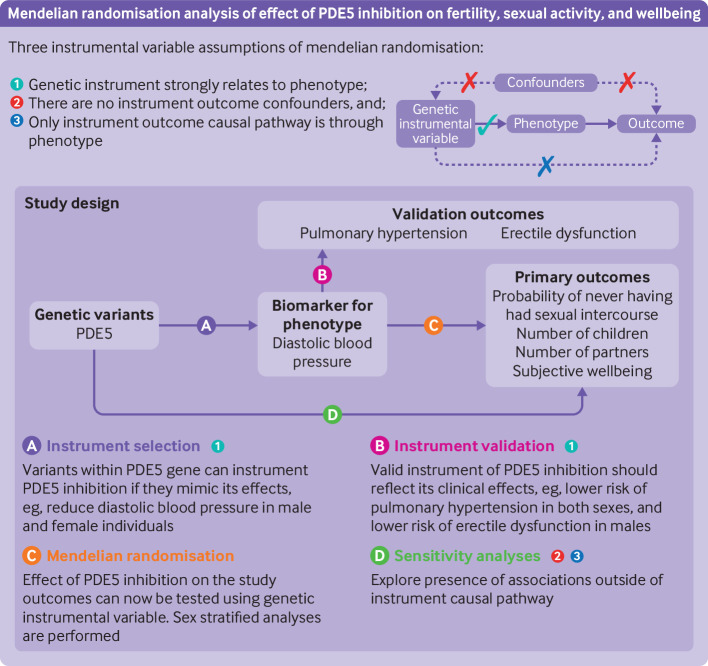
Schematic depiction of study design. PDE5=phosphodiesterase 5

We used cis*-*mendelian randomisation to explore the association of genetically proxied PDE5 inhibition with number of children fathered, number of sexual partners, probability of never having had sexual intercourse, and self-reported wellbeing. The main analyses were performed in male participants, with follow-up analyses performed in female participants to explore whether any identified associations were related to the presence of a penis (the erection of which might be facilitated by PDE5 inhibition). Figure 1 summarises the study design schematically. A glossary and guide to reading mendelian randomisation studies is published elsewhere.^6^

### Data sources

Estimates on the association between variant and blood pressure were extracted from a genome-wide association study of diastolic blood pressure.^11^ We selected diastolic blood pressure in preference to systolic blood pressure for the main analysis because PDE5 inhibition and resultant increased cyclic guanosine monophosphate activity induces vascular smooth muscle relaxation and vasodilation, which physiologically is expected to have a greater effect on diastolic blood pressure than systolic blood pressure.^12^ The diastolic blood pressure genome-wide association study meta-analysed data from 77 cohorts participating in the International Consortium for Blood Pressure and UK Biobank, comprising a total of 757 601 European participants of both sexes. Participating studies measured blood pressure using either manual or automated readings (mm Hg) and averaged the two readings when possible. Although the UK Biobank sample, which made up about 60% of the total study sample, adjusted for principal components, doing so was optional in the cohorts contributing to the International Consortium for Blood Pressure. All cohorts were adjusted for age, age^2^, sex, and body mass index, and the UK Biobank sample was additionally corrected for drug use. Further information on participant and genotype quality control checks can be found in the original publication.^11^ Genetic association estimates for diastolic blood pressure used in sensitivity analyses were obtained from the same study.

Variant-outcome associations were derived from UK Biobank, a large population cohort study of UK residents of predominantly European ancestry born between 1934 and 1971 and with about 500 000 participants.^13^ We conducted male participant only genome-wide association studies for two self-reported sexual behaviour outcomes (number of sexual partners, n=203 273; and probability of never having had sexual intercourse, n=211 840) and self-reported wellbeing (n=76 189). See the supplementary methods for details of the questionnaires used to obtain self-reported data. All of our sex specific UK Biobank genome-wide association studies were conducted using BOLT-LMM in the Medical Research Council Integrated Epidemiology Unit UK Biobank genome-wide association studies pipeline, adjusted for age, genotyping chip assay, and the first 10 principal components of ancestry.^14^ A full description of the pipeline methods, including filtering for quality control and imputation, can be found in the original publication.^14^ The pipeline by default excludes UK Biobank participants whose genetic sex differs from their reported gender. Variant-outcome information on number of children fathered was extracted from the Elsworth UK Biobank genome-wide association study (OpenGWAS identification No ukb-b-2227, n=209 872).^15^ Because about two thirds of the participants in the diastolic blood pressure genome-wide association study were from the UK Biobank, we expected substantial sample overlap between our phenotype and outcome samples, which could lead to bias if the variant-phenotype associations are statistically weak.

The supplementary methods provide details on data sources used for instrument selection, including expression quantitative trait loci. The expression quantitative trait loci and diastolic blood pressure data included both male and female participants.

### Statistical analysis

#### Instrument selection and validation

To select genetic variants for studying the effect of PDE5 inhibition, we considered single nucleotide polymorphisms within the PDE5 gene (GRCh37/hg19 chromosome 4 position 120 415 550–120 550 146) that associated with expression of the gene (ie, expression quantitative trait loci) in blood at genome-wide significance (P<5×10^−8^). To ensure that the variants used as instruments in mendelian randomisation are not highly correlated with each other, we then ranked them in order of the P values of their associations with diastolic blood pressure and pruned to linkage disequilibrium correlation r^2^<0.35 and distance threshold 10 000 kilobases. When accounting for correlation between variants, the inclusion of mildly correlated variants can improve power compared to selecting strictly independent variants. Mendelian randomisation estimators can, however, become unstable when variants are too highly correlated.^16^ The pruning r^2^ threshold was chosen to balance these two issues. As PDE5 inhibitors are used in the clinical management of erectile dysfunction and pulmonary hypertension,^2^ ^17^ we tested associations of our instrument for PDE5 inhibition with these two outcomes in positive control mendelian randomisation analyses.

#### Mendelian randomisation

To generate mendelian randomisation estimates, we estimated the Wald ratio for each genetic variant by dividing the variant-outcome association by the variant-diastolic blood pressure association. Standard errors for mendelian randomisation were estimated as the standard error of the variant-outcome association divided by the variant-phenotype association. Wald estimates for each variant were then meta-analysed with a multiplicative random effects model while using a linkage disequilibrium matrix corresponding to the European ancestry participants within the 1000G panel, as a source of reference to account for the correlation between variants.^16^ The Benjamini and Hochberg correction was used to account for multiple testing of the various outcomes.^18^ Sildenafil is a commonly used PDE5 inhibitor, with the 100 mg dose used for treating erectile dysfunction resulting in an approximate 5.5 mm Hg decrease in diastolic blood pressure.^19^ ^20^ To facilitate the clinical interpretation and contextualisation of our mendelian randomisation results, we scaled estimates to represent the diastolic blood pressure lowering effect of a 100 mg dose of sildenafil (ie, the main mendelian randomisation estimates are presented per 5.5 mm Hg reduction in diastolic blood pressure through genetically proxied PDE5 inhibition). Diastolic blood pressure was used as a biomarker to weight the effects of genetic variants, thus reflecting the effect of PDE5 inhibition, but it does not necessarily have to be the mechanism by which PDE5 inhibition is exerting its effect. Separate genome-wide association studies were used to obtain genetic associations with diastolic blood pressure and the considered outcomes. This two sample mendelian randomisation paradigm increases the available sample size for each of the genetic associations, thus increasing available statistical power. As a sensitivity analysis, mendelian randomisation was repeated using systolic blood pressure to weight the effects of genetic variants mimicking the effect of PDE5 inhibition, instead of diastolic blood pressure. To explore potential bias related to use of weak instruments, we repeated the main mendelian randomisation analysis excluding the variant with the weakest association with diastolic blood pressure, to assess whether the results were materially changed.

#### Colocalisation

One threat to the validity of cis-mendelian randomisation analyses is confounding by linkage disequilibrium. This occurs when a variant that associates with the phenotype is in linkage disequilibrium with a variant that associates with the outcome, thereby producing a spurious mendelian randomisation association. To explore the robustness of our results to confounding by linkage disequilibrium, we performed bayesian colocalisation using the Coloc statistical approach, between diastolic blood pressure and all outcomes for which a statistically significant mendelian randomisation association was identified. Coloc presents the evidence for five hypotheses: no causal variant for either trait, a causal variant for trait 1 but not trait 2, a causal variant for trait 2 but not trait 1, distinct causal variants underlying each trait, and a shared causal variant underlying both traits. A high posterior probability for the fifth hypothesis (>0.8) supports the presence of a shared causal variant underlying both traits, whereas a high posterior probability for the fourth hypothesis (>0.8) supports the presence of distinct causal variants underlying each trait, and thus indicates confounding by linkage disequilibrium in the corresponding mendelian randomisation association (which is also referred to as horizontal pleiotropy). In the presence of a statistically significant mendelian randomisation association that is not a false positive finding, if the posterior probability for both the fourth and fifth hypotheses are <0.8 this would suggest that the colocalisation analysis is likely underpowered to discriminate between whether the mendelian randomisation association is attributable to a shared causal variant or a confounding variant in linkage disequilibrium (ie, horizontal pleiotropy).

#### Replication in female patients

We replicated our primary mendelian randomisation analyses where we identified statistically significant associations using genome-wide association study summary data restricted to female participants, to investigate the penis dependence of our findings. In other words, we aimed to investigate whether effects related to the penis (and its propensity to become erect) may potentially explain any beneficial effects of PDE5 inhibition in male participants. The supplementary methods provide details on how we ran the outcome genome-wide association studies in female participants. Since these genome-wide association studies had a similar sample size to those performed in male participants, they should have similar statistical power.

#### Adjustment for potential bias related to pleiotropic associations

Horizontal pleiotropy occurs when a genetic variant influences the outcome through pathways other than the phenotype being studied, thereby violating a requisite assumption of mendelian randomisation (see supplementary methods). To explore potential horizontal pleiotropic effects, we searched PhenoScanner,^21^ a curated database of publicly available summary data from genome-wide association studies, for traits associated with the variants used to mimic PDE5 inhibition. The P value threshold for this was P<1×10^−5^, selected following Bonferroni correction for the number of traits in PhenoScanner.^21^ We then used two step cis*-*mendelian randomisation to adjust our mendelian randomisation estimates for any effect mediated by these traits (see supplementary figure S1). Two step cis*-*mendelian randomisation uses a two step mediation approach, similar to two step network mendelian randomisation, to adjust variant-outcome associations for potential pleiotropic pathways or confounding by linkage disequilibrium.^22^ ^23^ We additionally used two step cis*-*mendelian randomisation to adjust for body mass index to verify that its inclusion as a covariate in the diastolic blood pressure genome-wide association study had not induced collider bias.^24^ The supplementary methods provide further details, including the data sources used.

#### Software and preregistration

Mendelian randomisation analyses in this paper were run using the TwoSampleMR, TwoStepCisMR, MRPopTest, and meta R packages.^22^ ^25^ ^26^ ^27^ ^28^ Some data from genome-wide association studies were extracted from the OpenGWAS platform.^15^ The current study was not preregistered.

### Patient and public involvement

The first author (BW) has been prescribed PDE5 inhibitors for pulmonary hypertension. Although BW is currently childless, personal experience of the broad beneficial effects of PDE5 inhibitors on erectile or other physiological and psychological variables may or may not have inspired BW to undertake this study.

## Results

### Instrument selection and validation

After clumping, we identified five variants to serve as the genetic instrument for PDE5 inhibition (table 1). The lead variant predicted a 0.16 mm Hg lower diastolic blood pressure, and the average F statistic across all variants was 26, indicating low risk of weak instrument bias. The positive control analyses (see supplementary table 1) identified a mendelian randomisation association in the expected direction between genetically proxied PDE5 inhibition and erectile dysfunction (P=0.005) and pulmonary arterial hypertension (P<0.001).

**Table 1:** Single nucleotide polymorphism employed as instruments for phosphodiesterase 5 (PDE5) inhibition in the mendelian randomisation analyses

rsID	Chromosome	Position (hg19)	Effect allele	Other allele	Effect allele frequency		Diastolic blood pressure (mm Hg) (n=757 601)					eQTL* (n=31 684)
							β (mm Hg)	SE	P value	R^2^†	F statistic‡	P value
rs10050092	4	120532085	T	C	0.338		0.131	0.018	8.62E-13	6.73E-05	51	1.49E-18
rs12646525	4	120502461	C	T	0.785		0.095	0.021	6.74E-06	2.67E-05	20	1.46E-09
rs17355550	4	120416096	T	C	0.033		0.144	0.050	4.07E-03	1.09E-05	8	3.51E-08
rs66887589	4	120509279	C	T	0.478		0.161	0.017	1.83E-20	1.13E-04	86	1.87E-40
rs80223330	4	120423094	A	G	0.141		0.102	0.027	1.21E-04	1.95E-05	15	2.10E-34

eQTL=expression quantitative trait loci; SE=standard error.

* Genetic association estimates with protein expression quantitative trait loci were not available for these variants.

† Estimates the proportion of variance in the phenotype explained by the genetic variant.

‡ Measure of instrument strength.

### Main findings

**Fig 2:**
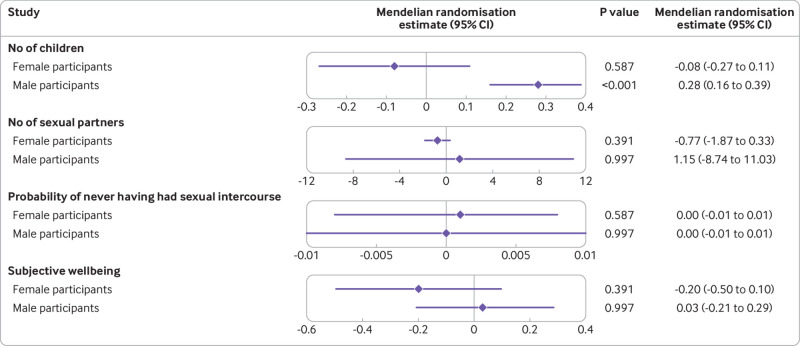
Mendelian randomisation results for genetically proxied phosphodiesterase 5 (PDE5) inhibition, scaled per 5.5 mm Hg lower diastolic blood pressure (which is the approximate effect of 100 mg sildenafil). Subjective wellbeing is measured in standard deviation units. CI=confidence interval

Table 2 shows the population characteristics for UK Biobank participants included in this study, and figure 2 presents the main results for mendelian randomisation. Scaled to the approximate diastolic blood pressure lowering effect of 100 mg of sildenafil (5.5 mm Hg), genetically proxied PDE5 inhibition was associated with male participants fathering on average 0.28 more children (95% confidence interval: 0.16 to 0.39 more children, false discovery rate corrected P<0.001). The second hypothesis—a causal variant for trait 1 but not trait 2, was the most likely (91%) in colocalisation analysis, suggesting that statistical power was insufficient to discern between whether the mendelian randomisation association was attributable to a shared causal variant or a variant in linkage disequilibrium (ie, horizontal pleiotropy).

**Table 2:** Descriptive characteristics of UK Biobank participants included in the main analysis

Phenotype (units)	Whole sample (n=462 918)				Male participants (n=211 840)				Female participants (n=251 078)		
	No of observations	Mean (SD) or No (%)	P value for association with GRS		No of observations	Mean (SD) or No (%)	P value for association with GRS		No of observations	Mean (SD) or No (%)	P value for association with GRS
Diastolic blood pressure (mm Hg)	432 519	82.2 (10.7)	<0.001*		198 168	84.0 (10.5)	<0.001*		234 351	80.577 (10.5)	<0.001*
Age (years)	462 918	56.7 (8.0)	0.574		211 840	57.0 (8.1)	0.217		251 078	56.6 (8.0)	0.697
Ever smoked	460 919	212 534 (46.1)	0.368		210 912	108 859 (51.6)	0.288		250 007	103 675 (41.5)	0.813
Self-reported ever having a depressive episode	149 463	81 963 (54.8)	0.694		65 098	28 613 (44.0)	0.154		84 365	53 350 (63.2)	0.323
Body mass index	461 368	27.4 (4.8)	0.007		211 081	27.9 (4.2)	0.017		250 287	27.0 (5.1)	0.125
Alcohol consumption monthly (units)	462 254	10.0 (11.7)	0.379		211 516	11.8 (12.2)	0.258		250 738	8.4 (11.1)	0.926
Years in education	307 836	16.7 (2.2)	0.338		138 145	16.7 (2.4)	0.626		169 691	16.6 (2.1)	0.054
Diabetes diagnosis	462 458	23 309 (5.0)	0.618		211 610	14 315 (6.8)	0.378		250 848	8994 (3.6)	0.075

GRS=phosphodiesterase 5 genetic risk score weighted by effect on diastolic blood pressure; SD=standard deviation.

All P values were derived from crude (ie, unadjusted) regression models. P value for the association between sex and the GRS was 0.909.

* After accounting for nine multiple tests, P values required a P<0.006 to be nominally significant with a 5% false positive rate.

We found no strong evidence of an association between genetically proxied inhibition of PDE5 and any of the other outcomes considered in male participants: number of sexual partners (1.15 (95% confidence interval −8.74 to 11.03), false discovery rate corrected P>0.99), probability of never having had sexual intercourse (additive increase in probability 0.00002 (95% confidence interval −0.01 to 0.01), false discovery rate corrected P>0.99), or self-reported wellbeing (standardised mean difference −0.03 (95% confidence interval −0.29 to 0.21), false discovery rate corrected P>0.99). As is apparent from the wide confidence interval, the null finding for number of sexual partners may be underpowered.

We did not find evidence of an association between genetically proxied PDE5 inhibition and number of children in female participants (β −0.08, 95% confidence interval −0.27 to 0.12). Supplementary table 2 presents the associations between genetically proxied PDE5 inhibition and the other outcomes in female participants. Little evidence of an association was found for any of the considered outcomes.

Sensitivity analyses using systolic blood pressure to weight mendelian randomisation estimates instead of diastolic blood pressure produced similar results (see supplementary table 3). We also found a similar result for the association between genetically predicted PDE5 inhibition and number of children for male participants when excluding the variant with the weakest association with diastolic blood pressure and using only the four variants with F statistics >10 (table 1) (0.28 more children, 95% confidence interval 0.16 to 0.40).

### Adjustment for potential bias related to pleiotropic associations

PhenoScanner identified 12 traits that associated (P<1×10^−5^) with at least one of the variants included in the PDE5 inhibition genetic instrument (see supplementary table 4). The conclusions of the main analysis did not change when adjusting for potential pleiotropic bias from these associations using two step cis-mendelian randomisation (see supplementary table 5). This suggests it is unlikely that the genetic variants explain any clinically relevant variation in health related outcomes that is not reported.

## Discussion

In this study, we investigated the association of genetically proxied PDE5 inhibition with measures of fertility, sexual behaviour, and wellbeing. We did not find evidence of an effect of PDE5 inhibition on number of sexual partners, probability of never having had sexual intercourse, or wellbeing in either male or female participants. We did, however, identify genetic evidence that lifelong PDE5 inhibition may increase the number of children had by male patients. Similar evidence was not identified in female patients, consistent with the notion that any effects of PDE5 inhibition on fertility in male patients may be attributable to penile mechanisms.^29^ ^30^ ^31^ ^32^ Erectile function is reduced in male patients with infertility, and it is estimated that more than one third of the male partners in couples seeking fertility treatment experience erectile dysfunction.^33^ ^34^ However, extra-penile mechanisms may also be at play. For example, a systematic review and meta-analysis found that oral PDE5 inhibitors improved sperm motility in male patients experiencing difficulties with infertility.^35^ Similarly, oral PDE5 inhibitors are associated with an increased proportion of morphologically normal sperm in male patients experiencing difficulties with infertility, and with improved sperm-oocyte binding.^35^

Although epidemiological evidence that PDE5 inhibition may have beneficial effects on fertility in female patients and reproductive outcomes exists,^36^ ^37^ ^38^ ^39^ ^40^ Cochrane systematic reviews have concluded that such evidence remains inadequate to derive any definitive policy recommendations.^41^ ^42^ However, a general limitation of population based studies is difficulty in appropriately quantifying fertility. The number of children people have is a function not only of their ability to have children but also of their desire to have children, among a range of other sociocultural factors. Fertility estimates can be artificially inflated by reproductive assistance, or artificially lowered by contraceptive use, unknown pregnancies, pregnancy termination, and miscarriages.

The potential implication of our research is that use of PDE5 inhibitors could improve fertility in male patients, particularly when this is related to erectile dysfunction. Further clinical study is, however, necessary to validate these findings. Consistent with our null finding in people who do not have penises, the effect of PDE5 inhibitors on fertility in male patients may be through effects on erectile function. Of relevance, random samples of general populations in the UK generally report higher age specific estimates of erectile dysfunction than the UK Biobank, where prevalence is less than 3%.^43^ Participants of UK Biobank may also have been undertreated when compared to a modern cohort. Since PDE5 inhibitors were widely approved for treating erectile dysfunction in the 1990s, most UK Biobank participants would likely already have attempted to have children before access to the drug class was widely available for erectile dysfunction. Mechanisms other than through erectile function may also be at play, including endocrine effects.

Because fertility is declining in many countries,^44^ ^45^ an intervention to improve sexual performance could help reverse this trend. We do not, however, recommend indiscriminate use of PDE5 inhibitors, which can have serious adverse effects, including loss of vision. Other potential implications of incorrect PDE5 inhibitor use might include hypotension and inappropriately timed erections. We emphasise that literal interpretations of mendelian randomisation estimates can be misleading, especially in instances where the causal estimate is likely to vary across the life course.^46^ ^47^ Thus, further research is required to estimate how PDE5 inhibitor use may affect fertility.

### Strengths and limitations of this study

This study leveraged cis*-*mendelian randomisation to investigate the causal effects of PDE5 inhibition. The novelty of our research question is strengthened by our analytical approach, which is more robust to the influence of confounding and reverse causation compared with traditional observational research methods. We showed the validity of our instrument with two positive controls, erectile dysfunction and pulmonary hypertension. Furthermore, use of two step cis-mendelian randomisation to adjust for potential biasing pleiotropic pathways provides further assurance that the mendelian randomisation assumptions are valid. By integrating biological knowledge to guide the study design, our findings are amenable to clinical contextualisation towards informing further research. This is paramount given the growing availability of large scale genetic association data, which may promote a temptation towards mis-specified application of mendelian randomisation for injudiciously contemplated research questions.

A potential limitation of our study is the apparent failure in the colocalisation analysis to support the association between genetically proxied PDE5 inhibition and number of children fathered. One possible explanation is limited statistical power, which may also have conceivably resulted in false negative findings for some of our other analyses. A second and important limitation is generalisability. Since genetic variants are inherited at conception and PDE5 inhibitors are typically used post-puberty, the mendelian randomisation estimates derived here may not be representative of PDE5 inhibitor use in practice. Furthermore, our study comprised participants of European ancestry and so we cannot be certain that our results would generalise to other populations. Finally, our mendelian randomisation model assumes that the effects of PDE5 inhibition are linear across the dose-response range. Of note, the genetic variants used as a proxy for the effect of PDE5 inhibition predicted less than a 1 mm Hg lower diastolic blood pressure, and thus our mendelian randomisation estimates may well not extrapolate to the effect of PDE5 inhibitors used in practice.

### Conclusions

We found genetic evidence to support the hypothesis that PDE5 inhibition may result in male patients fathering more children. This suggests that use of PDE5 inhibitors, and perhaps improved sexual performance in male patients more generally, might potentially help alleviate the declining fertility rates observed in many countries. However, further studies are required to confirm this, and we absolutely do not advocate indiscriminate use of PDE5 inhibitors—although relatively rare, PDE5 inhibitors can have harmful adverse effects.

**What is already known on this topic**

PDE5 inhibitors are a drug class commonly used for the treatment of erectile dysfunction, but their effects on fertility, sexual behaviour, and subjective wellbeing in male patients are not known
Drug target mendelian randomisation is a quasi-experimental method that uses genetic variants as instrumental variables for studying the effects of drug target perturbation

**What this study adds**

Evidence from drug target mendelian randomisation supports the potential for PDE5 inhibition to increase the number of children fathered by male patients, but with no evidence of such an effect in female patients
No strong evidence was found for PDE5 inhibitors affecting number of sexual partners, probability of never having had sexual intercourse, or subjective wellbeing in either male or female patients

## Data Availability

The data used in this study is publicly available from the Medical Research Council Integrated Epidemiology Unit OpenGWAS platform. The R code used in this study, and genome-wide association studies created for this project, are available from https://doi.org/10.17605/OSF.IO/MUERZ. This work was carried out using the computational facilities of the Advanced Computing Research Centre, University of Bristol (https://www.bris.ac.uk/acrc/). All genome-wide association studies summary data used in this research, including that created for the applied examples, will be made publicly available.

## Web extra

Extra material supplied by authors

Supplementary information: Additional methods, tables 1-5, figure 1, and STROBE-MR checklist

## Notes

Contributors: BW designed and implemented the study under the supervision of DG. All authors contributed to writing, editing, and revising the manuscript. BW is the guarantor of this study, accepts full responsibility for the work and conduct of the study, had access to the data, and controlled the decision to publish. The corresponding author attests that all listed authors meet authorship criteria and that no others meeting the criteria have been omitted.

The lead author (BW) affirms that this manuscript is an honest, accurate, and transparent account of the study being reported; that no important aspects of the study have been omitted; and that any discrepancies from the study as planned (and, if relevant, registered) have been explained.

Dissemination to participants and related patient and public communities: Upon publication, this work will be promoted by the authors on Twitter.

Provenance and peer review: Not commissioned; externally peer reviewed.
