# Supplementary material for: A drug target for erectile dysfunction to help improve fertility, sexual activity, and wellbeing: mendelian randomisation study
Source: BMJ. 2023 Dec 12;383:e076197. doi: 10.1136/bmj-2023-076197 (PMC10716676; doi:10.1136/bmj-2023-076197)
Supplement: Supplementary file 1 — Supplementary information: Additional methods, tables 1-5, figure 1, and STROBE-MR checklist [file woob076197.ww.pdf]

Supplement

Promoting erections to improve fertility, sexual activity and wellbeing: Mendelian randomisation study of a drug target for erectile dysfunction

Benjamin Woolf[1-3], Skanda Rajasundaram[4,5], Héléne T. Cronjé[6], James Yarmolinsky[2,7], Stephen Burgess[3], Dipender Gill[8]

[1]School of Psychological Science, University of Bristol, Bristol, UK

[2] Medical Research Council Integrative Epidemiology Unit, University of Bristol, Bristol, UK

[3]Medical Research Council Biostatistics Unit, University of Cambridge, Cambridge, UK

[4]Centre for Evidence-Based Medicine, University of Oxford, Oxford, UK

[5]Faculty of Medicine, Imperial College London, London, UK

[6]Department of Public Health, Section of Epidemiology, University of Copenhagen, Copenhagen, Denmark

[7]Population Health Sciences, University of Bristol, Bristol, UK

[8]Department of Epidemiology and Biostatistics, School of Public Health, Imperial College London, London, UK

Corresponding authors: Benjamin Woolf, School of Psychological Science, University of Bristol, Bristol, UK; benjamin.woolf@bristol.ac.uk.

Contents

Supplementary Methods 3

UK Biobank self-reported data 3

Data sources used for instrument selection and validation 3

Genome-wide association study in females 3

Further details on Mendelian randomization 4

Two-step *cis*-Mendelian randomization 4

References 5

Supplementary Tables 7

Supplementary Table 1. Positive control Mendelian randomization analysis results. 7

Supplementary Table 2. Mendelian randomization results for females. 8

Supplementary Table 3. Mendelian randomization results for males using systolic blood pressure to weight estimates. 9

Supplementary Table 4. Associations of instrument variants with traits in PhenoScanner. 10

Supplementary Table 5. Two-step *cis*-Mendelian randomization analysis results. 11

Supplementary Figure 12

Supplementary Figure S1. Directed acyclic graph for two-step *cis*-Mendelian randomization analysis. 12

STROBE-MR checklist 14

Supplementary Methods

UK Biobank self-reported data

In the UK Biobank (UKB), information on wellbeing (UKB identifier, ID 4526) was ascertained through a single question asked in follow-up in 2009: "In general how happy are you?", and then to choose either: “Extremely happy”, “very happy”, “Moderately happy”, “Moderately unhappy”, “Very unhappy”, “extremely unhappy”, “Do not know”, or “Prefer not to answer”. Information on number of sexual partners (UKB ID 2149) was ascertained through a single question asked at recruitment: "About how many sexual partners have you had in your lifetime?". The odds of being a virgin was estimated by recoding this question into a binary indicator so that people who had had one or more sexual partner were coded as 1 and leaving those who had had none as coded as zero.

Data sources used for instrument selection and validation

Expression quantitative trait loci (eQTL) data were taken from the 2018 eQTLGen Consortium genome-wide association study (GWAS) (OpenGWAS ID: eqtl-a-ENSG00000138735) of whole blood PDE5A expression (1). This study measured gene expression in 31,684 male and female participants of European ancestry.

Single-nucleotide (SNP) genetic associations with erectile dysfunction were estimated as the inverse-variance weighted meta-analysis of SNP effects form two GWASs. First, the Bovijn et al (2018) GWAS of erectile dysfunction (OpenGWAS ID: ebi-a-GCST006956). This GWAS had 6,175 European cases and 217,630 European controls (2). Second, we used the FinnGen round 8 GWAS of erectile dysfunction (OpenGWAS ID: finn-b-ERECTILE_DYSFUNCTION). This GWAS had 2,038 medical record inferred cases, and 157,478 controls. Finngen is a population cohort study of male and females of Finnish ancestry individuals living in Finland (3). Information of pulmonary arterial hypertension was extracted from the FinnGen round 8 GWAS of this trait in the OpenGWAS project (OpenGWAS ID: finn-b-I9_HYPTENSPUL). This GWAS had 213 medical record inferred cases, and 355,864 controls.

Genome-wide association study in females

The female only GWASs were conducted using the same methods as the male only GWASs.

To estimate variant-outcome associations in females, we used the female subset of sex-stratified GWASs for subjective wellbeing (N=89,815), number of sexual partners: (N=235,926), and the odds of being a virgin: 251,078 females). The estimate corresponding to fertility in females was the number of children they had birthed. This GWAS was performed using UKB data (OpenGWAS ID: ukb-b-1209, N=250,782) (4).

Further details on Mendelian randomization

Mendelian randomisation (MR) is a type of instrumental variable analysis that makes three core assumptions: 1) that the instrument (in this context a genetic variant) is strongly associated with the exposure, 2) that the instrument causes the outcome only via the exposure, and 3) that there is no instrument-outcome confounding. The F statistic of a variant describes the strength of its association with a trait. Bias due to violations of the first assumption of MR is inversely proportional to the F statistic for the variant-exposure association. We therefore evaluate this assumption by calculating the F statistic as the square of the variant-exposure association divided by the square of the standard error of this association. The second two assumptions cannot be proven empirically. In a *cis*-MR setting, which considers genetic variants at the gene for the protein being studied, the second assumption is more plausible since the exposure of interest, PDE5 inhibition, is very proximal to the gene, and there are therefore likely fewer pathways through which a pleiotropic effect could violate this assumption.

Two-step *cis*-Mendelian randomization

The product of coefficients methods of conducting a mediation analysis states that that the association between an exposure and an outcome mediated by a third variable is the association of the exposure with the third variable multiplied by the association of the third variable with the outcome. The difference of coefficients method for conducting a mediation analysis state that the mediated effect can be estimated as the total effect of the exposure on the outcome minus the direct effect of the exposure on the outcome (i.e., the effect not mediated by the third variable). It follows, that we can estimate the direct effect by subtracting the mediated effect from the total effect. Two-step *cis* MR leverages this to adjust variant-outcome estimates for potential sources of bias, by treating the source of bias as a mediator (5). For example, if the variant causes the outcome due to its association with a trait not related to the exposure, this effect can be accounted for by: 1)

estimating the variant-trait association (from a GWAS of the trait), 2) estimating the trait-outcome association (using MR), 3) estimating the variant-outcome association mediated by the trait by multiplying the estimated in 1) by the estimate in 2), and 4) adjusting the variant-outcome GWAS summary statistics by subtracting from them the estimates in 3).

We performed two-step *cis*-MR to adjust our main MR estimates for potentially confounding traits that associate with the variants employed as instruments. The 95% confidence intervals in both steps of the two-step *cis*-MR were estimated using bootstrap standard errors with 100,000 repetitions.

For the two-step *cis*-MR, GWAS summary data on platelet count (n = 350,474, OpenGWAS ID: ukb-d-30080_irnt), body mass index (BMI) (n = 461,460, OpenGWAS ID: ukb-b-19953), standing height (n = 461,950, OpenGWAS ID: ukb-b-10787), impedance of right arm (n = 454,826, OpenGWAS ID: ukb-b-7859), impedance of left arm (n=454,850, OpenGWAS ID: ukb-b-19379), impedance of whole body (n = 454,840, OpenGWAS ID: ukb-b-19921), impedance of left leg (n = 454,857, OpenGWAS ID: ukb-b-14068), impendence of right leg (n = 454,863, OpenGWAS ID: 'ukb-b-7376'), and white blood cell count (OpenGWAS ID: ieu-b-30) were extracted from existing UKB GWASs (4,6). Myeliod white cell count, granulocyte count, and sum basophil and neutrophil counts were extracted from the Astle et al (2016) GWAS of that trait (OpenGWAS ID: ebi-a-GCST004626, ebi-a-GCST004614, and ebi-a-GCST004620 respectively) (7). This GWAS had approximately 170,00 male and female participants, mostly recruited form UK Biobank sub-samples. Coronary artery disease summary data were taken from the van der Harst et al (2017) GWAS (OpenGWAS ID: ebi-a-GCST005195). This had 122,733 cases and 424,528 controls (male and female, of European ancestry) recruited from the UKB and CARDIoGRAMplusC4D (8).

References

1. Võsa U, Claringbould A, Westra HJ, Bonder MJ, Deelen P, Zeng B, et al. Large-scale cis- and trans-eQTL analyses identify thousands of genetic loci and polygenic scores that regulate blood gene expression. Nat Genet. 2021 Sep;53(9):1300–10.

2. Bovijn J, Jackson L, Censin J, Chen CY, Laisk T, Laber S, et al. GWAS Identifies Risk Locus for Erectile Dysfunction and Implicates Hypothalamic Neurobiology and Diabetes in Etiology. The American Journal of Human Genetics. 2019 Jan 3;104(1):157–63.

3. Kurki MI, Karjalainen J, Palta P, Sipilä TP, Kristiansson K, Donner K, et al. FinnGen: Unique genetic insights from combining isolated population and national health register data [Internet].

medRxiv; 2022 [cited 2022 Apr 14]. p. 2022.03.03.22271360. Available from: https://www.medrxiv.org/content/10.1101/2022.03.03.22271360v1

4. Elsworth B, Lyon M, Alexander T, Liu Y, Matthews P, Hallett J, et al. The MRC IEU OpenGWAS data infrastructure [Internet]. bioRxiv; 2020 [cited 2022 Mar 30]. p. 2020.08.10.244293. Available from: https://www.biorxiv.org/content/10.1101/2020.08.10.244293v1

5. Woolf B, Zagkos L, Gill D. TwoStepCisMR: A Novel Method and R Package for Attenuating Bias in cis-Mendelian Randomization Analyses. Genes. 2022 Sep;13(9):1541.

6. Neil B. Neale lab. [cited 2022 May 30]. UK Biobank. Available from: http://www.nealelab.is/uk-biobank

7. Astle WJ, Elding H, Jiang T, Allen D, Ruklisa D, Mann AL, et al. The Allelic Landscape of Human Blood Cell Trait Variation and Links to Common Complex Disease. Cell. 2016 Nov 17;167(5):1415-1429.e19.

8. van der Harst P, Verweij N. Identification of 64 Novel Genetic Loci Provides an Expanded View on the Genetic Architecture of Coronary Artery Disease. Circ Res. 2018 Feb 2;122(3):433–43.

Supplementary Tables

Supplementary Table 1. Positive control Mendelian randomization analysis results.

Outcome	Unit	Beta	se	p
Erectile dysfunction	Log odds per 1 mmHg increase in diastolic blood pressure	0.144	0.052	0.005
Pulmonary arterial hypertension		3.294	0.573	<0.001

Supplementary Table 2. Mendelian randomization results for females.

Outcome	Beta	Standard error	FDR p-value
Number of live births	-0.077	0.099	0.587
Number of sexual partners	-0.770	0.561	0.391
Probability of being a virgin	0.002	0.004	0.587
Subjective wellbeing (SD)	-0.198	0.154	0.391

Please note that these betas are scaled to the association of a 5.5mmHg decrease in PDE5 mediated blood pressure change on the respective outcomes. FDR: false discovery rate; SD: standard deviation units.

Supplementary Table 3. Mendelian randomization results for males using systolic blood pressure to weight estimates.

Outcome	Beta	Standard error	FDR p-value
Number of children	0.022	0.005	< 0.001
Number of sexual partners	0.113	0.518	0.983
Probability of being a virgin	$1x10^{-5}$	0.0006	0.983
Subjective wellbeing (SD)	0.005	0.007	0.983

Please note that these betas are scaled to the association of a 1 mmHg decrease in PDE5 mediated blood systolic pressure change on the respective outcomes. FDR: false discovery rate; SD: standard deviation units. The mean F statistic of this analysis was 25.

Supplementary Table 4. Associations of instrument variants with traits in PhenoScanner.

	rs80223330	rs12646525	rs17355550	rs66887589	rs10050092	openGWAS ID
Impedance of leg right		x			x	ukb-b-7376
Impedance of leg left		x			x	ukb-b-14068
Impedance of whole body					x	ukb-b-19921
Impedance of arm left					x	ukb-b-19379
Impedance of arm right					x	ukb-b-7859
Height					x	ukb-b-10787
Plateletcrit					x	ebi-a-GCST004607
Myeloid white cell count				x	x	ebi-a-GCST004626
Platelet count				x		ukb-d-30080
White blood cell count				x		ieu-b-30
Coronary artery disease	x			x		ebi-a-GCST005195
Granulocyte count				x		ebi-a-GCST004614
Sum basophil neutrophil counts				x		ebi-a-GCST004620

Crosses (x) denote associations at $p<1x10^{-5}$.

Supplementary Table 5. Two-step *cis*-Mendelian randomization analysis results.

Adjusting variable	OpenGWAS code	Effect estimate	Standard error
Body mass index	ukb-b-19953	0.22	0.03
Impedance of leg right	ukb-b-7376	0.22	0.03
Myeloid white cell count	ebi-a-GCST004626	0.21	0.03
Platelet count	ukb-d-30080	0.20	0.03
Coronary artery disease	ebi-a-GCST005195	0.21	0.03
Granulocyte count	ebi-a-GCST004614	0.21	0.03
Sum basophil neutrophil counts	ebi-a-GCST004620	0.21	0.03
Impedance of leg left	ukb-b-14068	0.22	0.03
Impedance of whole body	ukb-b-19921	0.23	0.03
Impedance of arm left	ukb-b-19379	0.24	0.03
Impedance of arm right	ukb-b-7859	0.24	0.03
height	ukb-b-10787	0.20	0.03
White blood cell count	ieu-b-30	0.21	0.03
Plateletcrit	ebi-a-GCST004607	0.21	0.03

Mendelian randomization estimates are for the outcome of number of children fathered and are scaled per 5.5mmHg diastolic blood pressure reduction (approximately that observed with 100mg sildenafil treatment).

Supplementary Figure

Supplementary Figure S1. Directed acyclic graph for two-step *cis*-Mendelian randomization analysis.

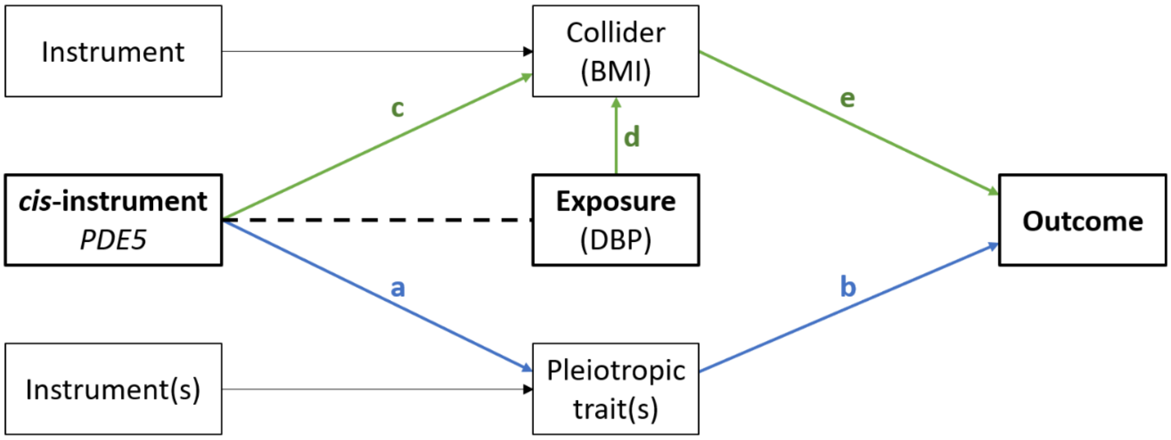

BMI: body mass index, DBP: diastolic blood pressure.

Accounting for pleiotropy (depicted in blue): We used PhenoScanner to identify potential pleiotropic traits, i.e., traits that are associated with our PDE5 instrument but not with the clinically plausible biomarker of its effect, i.e., diastolic blood pressure (DBP). Bias due to a pleiotropic effect can be quantified as a*b, i.e., the product of the instrument-pleiotropic trait association (a), and the Mendelian randomization (MR) estimate of the instrumented pleotropic trait on the outcome (b). We account for pleiotropy by subtracting a*b from our MR estimate.

Accounting for collider bias (depicted in green): MR estimates are generated using genome-wide association study (GWAS) summary statistics for the instrumented exposure (variant-exposure association) and the outcomes of interest (variant-outcome association). If the exposure GWAS reported estimates that were adjusted for covariates that were not adjusted for in the outcome GWAS, the MR model is subject to collider bias. In the case of our analyses, body mass index (BMI) was adjusted for in the GWAS of the exposure (DBP), but not in any of the outcome GWASs. Here, the bias relevant to the instrument-exposure association is proportional to c*d, i.e., the product of the instrument-BMI association (c) and the effect of the exposure (DBP) on the collider (BMI) (d). The relationship between the collider and the outcome is depicted as e. When e = 0, the exposure-outcome association will be incorrectly scaled, and the MR estimates will be biased. The bias in this case will not result in a detection of false positive findings as the z-statistic

of the MR estimate is unaffected by the (mis)scaling of the exposure. When $e \neq 0$, the MR z-statistic can be biased, but can be corrected by subtracting c*e from the instrument-outcome association, analogous to the pleiotropy setting.

When the collider impacts the SNP-outcome association, there is always a risk of inducing false positive results. A two-step procedure would be unlikely to remove bias in this instance. In our case, SNP-outcome collider bias is unlikely given that the SNP-outcome association did not adjust for BMI (or other heritable phenotypes).

STROBE-MR checklist

STROBE-MR checklist of recommended items to address in reports of Mendelian randomization studies[1 2]

Item No.	Section		Checklist item	Page No.	Relevant text from manuscript
1	**TITLE and ABSTRACT**		Indicate Mendelian randomization (MR) as the study's design in the title and/or the abstract if that is a main purpose of the study	1	Title page
	**INTRODUCTION**				
2	**Background**		Explain the scientific background and rationale for the reported study. What is the exposure? Is a potential causal relationship between exposure and outcome plausible? Justify why MR is a helpful method to address the study question	4-5	Introduction section
3	**Objectives**		State specific objectives clearly, including pre-specified causal hypotheses (if any). State that MR is a method that, under specific assumptions, intends to estimate causal effects	5	Final paragraph
	**METHODS**				
4	**Study design and data sources**		Present key elements of the study design early in the article. Consider including a table listing sources of data for all phases of the study. For each data source contributing to the analysis, describe the following:	6	Study design
		a)	Setting: Describe the study design and the underlying population, if possible. Describe the setting, locations, and relevant dates, including periods of recruitment, exposure, follow-up, and data collection, when available.	6-7	Data sources section
		b)	Participants: Give the eligibility criteria, and the sources and methods of selection of participants. Report the sample size, and whether any power or sample size calculations were carried out prior to the main analysis	6-7	Data sources section
		c)	Describe measurement, quality control and selection of genetic variants	6-7	Data sources section
		d)	For each exposure, outcome, and other relevant variables, describe methods of assessment and diagnostic criteria for diseases	7	Instrument selection section
		e)	Provide details of ethics committee approval and participant informed consent, if relevant	NA	NA

5	**Assumptions**	Explicitly state the three core IV assumptions for the main analysis (relevance, independence and exclusion restriction) as well assumptions for any additional or sensitivity analysis	Supplementary methods	
6	**Statistical methods: main analysis**	Describe statistical methods and statistics used	7-8	Statical analysis section
	a)	Describe how quantitative variables were handled in the analyses (i.e., scale, units, model)	6-7	Data sources section
	b)	Describe how genetic variants were handled in the analyses and, if applicable, how their weights were selected	6-7	Statical analysis section
	c)	Describe the MR estimator (e.g. two-stage least squares, Wald ratio) and related statistics. Detail the included covariates and, in case of two-sample MR, whether the same covariate set was used for adjustment in the two samples	6-7	Statical analysis section/supplement
	d)	Explain how missing data were addressed	NA	NA
	e)	If applicable, indicate how multiple testing was addressed	7	Statical analysis section
7	**Assessment of assumptions**	Describe any methods or prior knowledge used to assess the assumptions or justify their validity	Supplement	
8	**Sensitivity analyses and additional analyses**	Describe any sensitivity analyses or additional analyses performed (e.g. comparison of effect estimates from different approaches, independent replication, bias analytic techniques, validation of instruments, simulations)	7-9	Last 3 sub section of Statistical analysis section
9	**Software and pre-registration**			
	a)	Name statistical software and package(s), including version and settings used	9	Eponymous section
	b)	State whether the study protocol and details were pre-registered (as well as when and where)	9	Eponymous section
	**RESULTS**			
10	**Descriptive data**			
	a)	Report the numbers of individuals at each stage of included studies and reasons for exclusion. Consider use of a flow diagram	Figure 1	

		b)	Report summary statistics for phenotypic exposure(s), outcome(s), and other relevant variables (e.g. means, SDs, proportions)	NA	
		c)	If the data sources include meta-analyses of previous studies, provide the assessments of heterogeneity across these studies	NA	
		d)	For two-sample MR:<br>i. Provide justification of the similarity of the genetic variant-exposure associations between the exposure and outcome samples<br>ii. Provide information on the number of individuals who overlap between the exposure and outcome studies	NA	These are provided in the preprint https://www.medrxiv.org/content/ 10.1101/2023.03.27.23287822v1
11	**Main results**				
		a)	Report the associations between genetic variant and exposure, and between genetic variant and outcome, preferably on an interpretable scale	Table 1	
		b)	Report MR estimates of the relationship between exposure and outcome, and the measures of uncertainty from the MR analysis, on an interpretable scale, such as odds ratio or relative risk per SD difference	11	Main results
		c)	If relevant, consider translating estimates of relative risk into absolute risk for a meaningful time period	NA	
		d)	Consider plots to visualize results (e.g. forest plot, scatterplot of associations between genetic variants and outcome versus between genetic variants and exposure)	Figure 2	
12	**Assessment of assumptions**				
		a)	Report the assessment of the validity of the assumptions	11-12	Results section
		b)	Report any additional statistics (e.g., assessments of heterogeneity across genetic variants, such as I^2, Q statistic or E-value)	NA	
13	**Sensitivity analyses and additional analyses**				
		a)	Report any sensitivity analyses to assess the robustness of the main results to violations of the assumptions	11-12	Results section

		b)	Report results from other sensitivity analyses or additional analyses	NA	
		c)	Report any assessment of direction of causal relationship (e.g., bidirectional MR)	NA	
		d)	When relevant, report and compare with estimates from non-MR analyses	NA	
		e)	Consider additional plots to visualize results (e.g., leave-one-out analyses)	Figure 2	
	**DISCUSSION**				
14	**Key results**		Summarize key results with reference to study objectives	13-14	Key findings and interpretation
15	**Limitations**		Discuss limitations of the study, taking into account the validity of the IV assumptions, other sources of potential bias, and imprecision. Discuss both direction and magnitude of any potential bias and any efforts to address them	14-15	Strength and limitations
16	**Interpretation**				
		a)	Meaning: Give a cautious overall interpretation of results in the context of their limitations and in comparison with other studies	P12-15	
		b)	Mechanism: Discuss underlying biological mechanisms that could drive a potential causal relationship between the investigated exposure and the outcome, and whether the gene-environment equivalence assumption is reasonable. Use causal language carefully, clarifying that IV estimates may provide causal effects only under certain assumptions	P12-15	
		c)	Clinical relevance: Discuss whether the results have clinical or public policy relevance, and to what extent they inform effect sizes of possible interventions	P12-15	
17	**Generalizability**		Discuss the generalizability of the study results (a) to other populations, (b) across other exposure periods/timings, and (c) across other levels of exposure	P12-15	
	**OTHER INFORMATION**			16	
18	**Funding**		Describe sources of funding and the role of funders in the present study and, if applicable, sources of funding for the databases and original study or studies on which the present study is based	16	
19	**Data and data sharing**		Provide the data used to perform all analyses or report where and how the data can be accessed, and reference these sources in the article. Provide the statistical code	16	

		needed to reproduce the results in the article, or report 1whether the code is publicly accessible and if so, where	
20	**Conflicts of Interest**	All authors should declare all potential conflicts of interest	16

This checklist is copyrighted by the Equator Network under the Creative Commons Attribution 3.0 Unported (CC BY 3.0) license.

1. Skrivankova VW, Richmond RC, Woolf BAR, Yarmolinsky J, Davies NM, Swanson SA, et al. Strengthening the Reporting of Observational Studies in Epidemiology using Mendelian Randomization (STROBE-MR) Statement. JAMA. 2021;under review.

2. Skrivankova VW, Richmond RC, Woolf BAR, Davies NM, Swanson SA, VanderWeele TJ, et al. Strengthening the Reporting of Observational Studies in Epidemiology using Mendelian Randomisation (STROBE-MR): Explanation and Elaboration. BMJ. 2021;375:n2233.
